# Mesocosm experiments to better understand hydrocarbon half-lives for oil and oil dispersant mixtures

**DOI:** 10.1371/journal.pone.0228554

**Published:** 2020-01-31

**Authors:** Maya E. Morales-McDevitt, Dawei Shi, Anthony H. Knap, Antonietta Quigg, Stephen T. Sweet, Jose L. Sericano, Terry L. Wade

**Affiliations:** 1 Geochemical and Environmental Research Group, Texas A & M University, College Station, Texas, United States of America; 2 Department of Oceanography, Texas A & M University, College Station, Texas, United States of America; 3 Department of Marine Biology, Texas A & M University at Galveston, Galveston, Texas, United States of America; University of Siena, ITALY

## Abstract

Concerns on the timing and processes associated with petroleum degradation were raised after the use of Corexit during the Deepwater Horizon oil spill. There is a lack of understanding of the removal of oil associated with flocculate materials to the sediment. Mesocosm studies employing coastal and open-ocean seawater from the Gulf of Mexico were undertaken to examine changes in oil concentration and composition with time. The water accommodated fractions (WAF) and chemically enhanced WAF (CEWAF) produced using Macondo surrogate oil and Corexit were followed over 3–4 days in controlled environmental conditions. Environmental half-lives of estimated oil equivalents (EOE), polycyclic aromatic hydrocarbons (PAH), n-alkanes (C10-C35), isoprenoids pristane and phytane, and total petroleum hydrocarbons (TPH) were determined. EOE and PAH concentrations decreased exponentially following first-order decay rate kinetics. WAF, CEWAF and DCEWAF (a 10X CEWAF dilution) treatments half-lives ranged from 0.9 to 3.2 days for EOE and 0.5 to 3.3 days for PAH, agreeing with estimates from previous mesocosm and field studies. The aliphatic half-lives for CEWAF and DECWAF treatments ranged from 0.8 to 2.0 days, but no half-life for WAF could be calculated as concentrations were below the detection limits. Biodegradation occurred in all treatments based on the temporal decrease of the nC_17_/pristane and nC_18_/phytane ratios. The heterogeneity observed in all treatments was likely due to the hydrophobicity of oil and weathering processes occurring at different rates and times. The presence of dispersant did not dramatically change the half-lives of oil. Comparing degradation of oil alone as well as with dispersant present is critical to determine the fate and transport of these materials in the ocean.

## Introduction

The fate, transport, and transformation of oil components in the water column depend on complex interactions of processes called weathering which includes dissolution, dispersion, evaporation, photo-oxidation, sorption onto particulate matter (e.g., association of oil with marine snow), and biodegradation [1]. In the case of the Macondo crude oil released during the Deepwater Horizon incident, the relative importance weathering processes were not well understood.

One experimental approach for investigating the behavior of oil components in a controlled environment is the use of enclosed marine ecosystems, termed mesocosms [2, 3, 4]. Mesocosms consist of partially or fully enclosed containers providing an experimental system for researchers that partly mimic natural ecosystems [5] while allowing controlled replicate experiments that include biotic effects [3, 6, 7]. Through mesocosm experiments, a more complete and quantitative understanding of the fate of hydrocarbons as well as their ecological effects is possible [8]. Vertical transport of petroleum hydrocarbons and their incorporation to bottom sediments [8, 4, 6] and the fate of the water-soluble fraction of oil and its effect on marine coastal organisms [9, 10] have been investigated using this approach.

The Deepwater Horizon oil spill resulted in the formation of profuse flocculent marine oil snow (MOS) [11, 12, 13]. Passow et al. [11] hypothesized the MOS was formed *in situ* by the interaction of microbes and oil and removed from the water column by sedimentation. Daly et al. [14] reviewed marine oil snow sedimentation and flocculant accumulation (MOSSFA) processes while Quigg et al. [15] summarized the plethora of marine snow materials produced by microbes. Petroleum degrading microbes are associated with marine snow [16] and analyses of sediments for petroleum hydrocarbons report that oil beyond 5 km from the Deepwater Horizon wellhead had undergone extensive weathering of hydrocarbons below n-C25 [17]. Alkane measurements taken after the spill in water and sediments show a decreasing n-C17/pristane ratio over time, indicating active bacterial biodegradation [18, 17]. The chemical composition changes of oil can be used to assess biodegradation patterns and processes [19, 1]. Macondo oil is a light crude oil [18] with both n-alkanes and polycyclic aromatic hydrocarbons (PAH) expected to decrease as a function of time. While the present study reported here focuses on oil chemistry, other related studies have reported on the preparation of the oil for the mesocosms [7], the role of bacteria [16], and phytoplankton [20] in addition to the various forms of MOS [21, 22] and its formation [23].

The objective of this research was to monitor temporal changes in oil composition in replicate mesocosms performed with open-ocean (M3) and coastal waters (M4) from the Gulf of Mexico, with either oil or oil plus dispersant introduced into the system. The use of large (~80 L) mesocosms allowed sufficient sample material to be collected for companion studies (see above). Specific elements monitored include estimated oil equivalents (EOE), total petroleum hydrocarbons (TPH), n-alkanes, PAHs, and the more recalcitrant isoprenoid hydrocarbons pristane and phytane over the course of short-term (3 to 4 days) mesocosm experiments that were performed sequentially. Comparing degradation of oil alone as well as with dispersant present is critical to determine the fate and transport of these materials in the ocean.

## Methods and materials

### 2.1. Water collection

The surface seawater used for “open-ocean” Mesocosm M3 (salinity 32 PSU) was collected on July 8^th^, 2016 using the *R/V Trident* adjacent to the Flower Garden Banks National Marine Sanctuary Area (27° 53’ N; 94° 02’ W) located approximately 180 km south of Galveston, Texas. The “coastal” Mesocosm M4 surface seawater (salinity 28 PSU) was collected on the *R/V Trident* from the Texas coastline on July 14^th^, 2016, (29° 22' N; 93° 23’ W) approximately 120 km east of Galveston, Texas and closer to shore compared to the seawater for M3. Seawater was collected ~24 hr before the start of the production of WAF and CEWAF. No specific permissions were required for these locations and activities and no endangered or protected species were involved as we simply collected surface water seawater from this location.

### 2.2. Water accommodated fractions of oil

As demand for the MC252 (Macondo oil) exceeded supply, surrogate oils for research purposes were made available by BP [24]. Oil from the Marlin platform nearby to the Macondo well, is similar to MC252 having a specific gravity of 0.86, and similar chemical composition (e.g. n-alkane and polycyclic aromatic hydrocarbon distribution) and aquatic toxicity [24, 25, 7]. A detailed comparison between the Macondo source oil and Marlin Platform oil is found in Worton et al. [24].

The Chemical Response to Oil Spills: Ecological Effects Research Forum (CROSERF) method for the generation of relatively small volumes (up to a few liters) of a water-accommodated fraction (WAF) of oil [26] has been used for many studies. The larger volume needed for the studies described here required the development of baffled recirculation tanks (BRT) to prepare these bigger volumes (100’s of L) for these mesocosm studies [27, 7]. These BRTs were used to prepare WAF, a chemically enhanced water accommodated fraction (CEWAF) of oil and a diluted (10-fold) CEWAF (DCEWAF). Flow was generated by a PTFE-diaphragm pump that re-circulated the seawater at 350 ml min^-1^ [7]. Additionally, an electric stirrer was used at rates no higher than 200 rpm to avoid creating a vortex using low energy mixing [26]. The WAF and CEWAF produced contain both dissolved and particulate (including oil globules of varying sizes) hydrocarbons [7].

For the preparation of WAF, 25 ml of surrogate oil was added directly to the BRT. For CEWAF, 25 ml of oil was premixed with the dispersant Corexit in a 20:1 the ratio to oil recommended by the US Environmental Protection Agency. In order to produce DCEWAF, CEWAF was prepared then diluted 10-fold, with the collected seawater [7]. The BRT produced concentrations of oil ranging from 0.2 to 1 mg/L for WAF, 2–8 mg/L for DCEWAF and 39 to 80 mg/L for CEWAF. These concentrations are similar to concentrations reported by Knap et al. [27]. Corexit only mesocosms were not considered because during an oil spill response, it is not added to seawater if oil is not present. Controls provided background information on the hydrocarbon composition of the seawater prior to the preparation of WAF and CEWAF is provided in the control tanks.

### 2.3. Mesocosms

For M3 and M4, triplicate mesocosms were used for each treatment (i.e., WAF, CEWAF, DCEWAF and control; 12 total tanks) containing 90 L of water each. Fluorescent lights were positioned at the front of the mesocosm tanks (12 h light/12 h dark; 50–80 μmol photons m^-2^ s^-1^) at room temperature (~21°C). Nutrients solutions were prepared using the Guillard and Ryther [28] recipe for f/2 media but diluted to f/20 before addition. Final concentrations of nutrient stocks prepared were: NaNO_3_ (8.82 x 10^−4^ M), NaH_2_PO_4_.H_2_O (3.62 x 10^−5^ M) and Na_2_SiO_3_.9H_2_O (1.06 x 10^−4^ M), respectively. These solutions were added at time zero and vigorously stirred. All mesocosm tanks had PTFE spigots 10 cm above the bottom for sample collection to avoid sampling the water surface or breaking marine snow particles.

### 2.4. Estimated oil equivalents

The EOE concentrations [7] in the WAF, CEWAF and DCEWAF tanks were measured at ~24 hr time intervals. For each measurement, 5, 10 or 20 ml of seawater was collected from each mesocosm and extracted with 5 ml of dichloromethane (DCM). It was necessary to increase the collection volume over time to compensate for decreasing EOE concentration. Approximately 2 ml of the DCM fraction was transferred into a 10 mm quartz cuvette and analyzed by fluorometry using a Horiba Scientific Aqualog fluorometer. Optimum wavelengths for EOE surrogate oil were found to be λ_Ex_: 260 nm and λ_Em_: 372 nm based on the fluorescence maximum obtained by scanning excitation and emission wave lengths from 200 to 500 nm. Detection limits for EOE was 0.07 mg/L. This extraction method from Wade et al. [29] includes both particulate and dissolved oil components. A calibration curve was generated using the Macondo surrogate oil prepared at five concentrations ranging from 0.086 mg/L to 4.3 mg/L.

### 2.5. Water sample extraction

Samples (1–3.5L) were collected in amber bottles with Teflon lined screw caps at the start (time zero) and every 24 hours from each of the triplicate treatment tanks. DCM was added (~20ml) immediately as a preservative. Prior to the extraction, PAH surrogates (d_8_-naphthalene, d_10_-acenaphthene, d_10_-phenanthrene, d_12_-chrysene and d_12_-perylene) and aliphatic surrogate standards (deuterated nC_12_, nC_20_, nC_24_ and nC_30_) were added to the water samples. Water samples were extracted in separatory funnels two or three times using 70 to 100 ml of DCM each time. The DCM was reduced in volume and exchanged into hexane [29]. The 2 mL hexane aliquot was transferred to silica gel/alumina columns for purification. Prior to transferring the samples, columns were packed with a plug of glass wool, 2 cm of sand, 10 g of alumina, 20 g of silica gel and a thin layer of sodium sulfate. Columns were conditioned with DCM followed by pentane. Hydrocarbons were eluted with 200 mL of a 1:1 pentane/dichloromethane solution. Samples were collected in 250 ml flat bottom flasks, evaporated and exchanged to 1 ml of hexane carefully to prevent the samples going dry [29, 25]. Samples were spiked with PAH (d_10_-fluorene and d_12_-benzo[a]pyrene) and aliphatic (deuterated *n*-C_16_) recovery standards.

### 2.6. Total petroleum analysis

Aliphatic hydrocarbons and TPH were analyzed on an Agilent 7890 gas chromatograph with a flame ionization detector (GC/FID) [29] with an Agilent DB-5MS fused capillary column (30 m long x 0.25 mm I.D. with a 0.25μ film thickness). The oven program was set at 60°C for 1 min, then 6°C/min to 300°C and held for 10 min. The n-alkanes ranging from *n*-C10 to *n*-C35, and the isoprenoids pristane and phytane were quantitated using relative response factors calculated from the response of the analyte in calibration standards. Total resolved (TR), unresolved complex mixture (UCM) and total petroleum hydrocarbons (TPH) concentrations were calculated using an average of the relative response factors for all n-alkanes and isoprenoids present in the calibration standard and the relevant areas. The UCM is composed of thousands of hydrocarbons, which are not resolved as peaks from each other (co-elute), they produce a hump in the gas chromatogram. Total resolved hydrocarbons is the sum of the area from all peaks from the retention time of *n*-C10 to the retention time for *n*-C35 with the surrogate and internal standard areas removed. TPH is the total integrated area above a straight line starting at the retention time of *n*-C10 to *n*-C35 with the surrogate and internal standard areas removed by subtraction of the total integrated area from a blank to correct for any baseline rise. UCM concentration is the difference between TPH and TR [29]. Detection limits for petroleum compounds were as follows; PAHs, n-alkanes, pristane and phytane at 2 ng/L, TPHs at 0.2 ug/L, and total resolved and UCM at 50 ug/L.

### 2.7. Polycyclic aromatic hydrocarbon analysis

PAHs were analyzed on a Hewlett-Packard 6890 gas chromatograph (GC) coupled with a Hewlett-Packard 5973 mass selective detector. A laboratory reference sample (diluted oil sample) was analyzed with each batch of samples to confirm GC/MS/SIM system performance and calibration. Instrumental calibrations were checked by injection of a mid-level calibration solution. Separation of PAHs was accomplished with a DB-5 MS fused silica capillary column (30 m×0.25 mm i.d., 0.25 μm film thickness, J&W Scientific). The oven temperature was programmed to increase from an initial temperature of 60°C to 150°C at 15°C min^-1^, then 5°C min^−1^ to 220°C, and finally at 10°C min^−1^ to a final temperature of 300°C with a final holding time of 10 min. The PAHs were identified based on the comparison of the retention time and mass spectrum of selected ions with the calibration standards. Alkylated PAH were quantitated based response of the parent PAH compound (e.g. naphthalene response factor was used to determine naphthalene with 1–4 substituted carbons).

## Results and discussion

### 3.1. Estimated oil equivalents

EOE concentrations of the WAF, DCEWAF and CEWAF for M3 and M4 are provided in Table 1. Average triplicate WAF EOE concentrations at time 0 for M3 and M4 were 0.74 (70% RSD) and 0.29 (10% RSD) mg/L, respectively (Table 1). The high percent RSD demonstrates the variability inherent in the production of large volumes of WAF at different times and between the three replicate mesocosms. Similar variability has been reported by other studies [3, 27, 7]. Average triplicate DCEWAF EOE concentrations at time 0 for M3 and M4 were 6.17 (22% RSD) and 8.13 (12% RSD) mg/L, respectively (Table 1). Average triplicate CEWAF EOE concentrations at time 0 for M3 and M4 were 39.1 (2% RSD) and 81.1 (25% RSD) mg/L, respectively (Table 1). The DCEWAF for M3 as a tenfold dilution on the CEWAF was expected to have a concentration of ~ 4 mg/l but averaged 6.17 mg/L this is likely due to heterogeneity of the starting CEWAF. Starting CEWAF concentrations were similar to a worst-case scenario for a spill [30, 31, 32, 33, 34, 7].

**Table 1:** Change in oil composition during mesocosm experiments. Estimated oil equivalents (EOE), total petroleum hydrocarbons (TPH), total resolved (TR), unresolved complex mixture (UCM) and total alkanes concentrations (mg/L). The percent relative standard deviation (%RSD) and the half-lives based on changes after 3 days are also included.

		Time (d)	EOE		PAH		Alkanes		Resolved		UCM		TPH	
			mg/L	%RSD	mg/L	%RSD	mg/L	%RSD	mg/L	%RSD	mg/L	%RSD	mg/L	%RSD
Mesocosm 3	WAF	0	0.74	70.4	0.0536	3.3	ND		ND		ND		ND	
		1	0.43	51.9	0.0066	1.1	ND		ND		ND		ND	
		2	0.30	48.9	0.0005	33.7	ND		ND		ND		ND	
		3	0.15	58.3	0.0007	42.2	ND		ND		ND		ND	
		4	0.07	74.9	0.0009	39.2	ND		ND		ND		ND	
		Half Life (d)	1.2		0.5		ND		ND		ND		ND	
	DCEWAF	0	6.17	21.7	0.0937	1.2	0.36	7.1	0.60	9.9	2.19	6.8	2.79	6.5
		1	5.65	5.7	0.0698	2.9	0.14	11.3	0.24	18.5	2.06	50.5	2.30	46.2
		2	4.21	14.5	0.0167	6.2	0.03	6.7	0.03	18.4	0.88	24.4	0.91	23.7
		3	3.20	25.3	0.0117	8.6	0.03	4.6	0.14	3.6	1.48	14.7	1.62	13.7
		4	2.71	6.0	0.0073	17.3	0.02	8.4	0.09	2.9	1.23	8.7	1.32	8.1
		Half Life (d)	3.2		1.0		0.8		1.4		5.3		3.8	
	CEWAF	0	39.07	2.0	0.3230	52.8	1.34	63.3	2.89	68.9	6.48	56.7	9.37	60.0
		1	24.20	11.6	0.2210	10.2	0.78	11.4	1.45	42.1	6.00	14.8	7.45	8.8
		2	19.63	12.9	0.1910	4.5	0.51	3.9	1.54	19.9	4.73	11.7	6.27	13.3
		3	12.39	15.9	0.1640	6.5	0.47	52.5	1.41	54.9	5.01	51.5	6.42	52.1
		4	8.21	31.3	0.0730	31.2	0.75	16.9	1.77	20.5	7.51	17.3	9.28	14.3
		Half Life (d)	1.8		3.1		2.0		2.9		8.1		5.5	
Mesocosm 4	WAF	0	0.29	9.3	0.0523	5.1	ND		ND		ND		ND	
		1	0.14	29.4	0.0015	48.1	ND		ND		ND		ND	
		2	0.09	15.9	0.0014	31.4	ND		ND		ND		ND	
		3	0.03	29.5	0.0010	22.4	ND		ND		ND		ND	
		4	ND		ND		ND		ND		ND		ND	
		Half Life (d)	0.9		0.5		ND		ND		ND		ND	
	DCEWAF	0	8.13	11.9	0.1020	13.7	0.31	81.7	0.82	62.8	1.72	32.9	2.54	40.0
		1	5.40	16.8	0.0794	13.5	0.04	49.4	0.17	131.3	0.96	1.6	1.13	10.3
		2	4.00	26.1	0.0332	83.0	0.03	60.5	0.12	53.7	1.17	59.1	1.29	57.9
		3	1.84	60.7	0.0154	20.3	0.02	171.4	0.11	169.2	1.09	153.9	1.20	159.0
		4	ND		ND		ND		ND		ND		ND	
		Half Life (d)	1.4		0.9		0.8		1.0		4.6		2.8	
	CEWAF	0	81.06	25.3	0.4538	1.5	4.17	80.3	10.06	79.6	19.67	55.0	29.73	62.9
		1	38.77	9.2	0.3503	7.4	1.55	31.3	4.20	30.4	11.66	8.7	15.86	13.5
		2	33.17	14.0	0.2721	5.3	1.46	85.3	4.12	85.2	12.56	74.5	16.69	77.2
		3	19.83	6.6	0.2422	10.1	0.37	22.9	1.33	23.4	7.47	13.2	8.81	14.8
		4	ND		ND		ND		ND		ND		ND	
		Half Life (d)	1.5		3.3		0.9		1.0		2.1		1.7	

### 3.2. TPH, alkanes, TR and UCM hydrocarbons

The concentration of TPH, alkanes, TR (total resolved) and UCM (unresolved complex mixture) hydrocarbons during M3 and M4 studies decreased with time in all treatments (Table 1). TPH is a common measurement performed during oil spills to assess the total concentration of nonvolatile high molecular weight (HMW) hydrocarbons in seawater [25, 1]. The average TPH concentrations for the DCEWAF and CEWAF at the beginning of M3 were 2.79 and 9.37 mg/L respectively. The average TPH concentrations for the DCEWAF and CEWAF at the beginning of M4 were 2.54 and 29.7 mg/L respectively. TPH in waters samples analyzed during and after the Deepwater Horizon (DWH) oil spill ranged from <0.001 to 7,200 mg/L and only 5% were above 0.25 mg/L [25]. TPH in the experiments described here are in the upper range of these concentrations. The TPH is the sum of the TR plus the UCM. The n-alkanes and pristane and phytane are part of the TR. The half-lives estimated for these parameters all are in the order of alkanes<TR<TPH<UCM (Table 1). So while all the concentrations of resolved peaks are decreasing the UCM is decreasing slower (Table 1). This result compares well to other studies of hydrocarbon weathering [1]. In many sediment studies the majority of hydrocarbons remaining as a UCM [8, 17].

### 3.3. Normal alkane ratios

The n-alkanes in oil are saturated, straight chain hydrocarbons with single bonds that are easily biodegraded by oxidation of the terminal carbon atom [1, 35, 36]. In the present study, the composition of the Macondo surrogate oil was characterized in detail using GC/FID. The variability in our analyses (not reported) for the more volatile compounds was similar to that reported in the Gulf of Mexico Research Initiative Hydrocarbon Intercalibration Experiment with over thirty participating laboratories [37]. The DWH oil and the Macondo surrogate oil, are typical light Louisiana crude oils composed of saturated n-alkanes, PAH and alkylated PAH [18]. Higher abundances of the shorter chain alkanes in the surrogate oil makes this oil subject to rapid weathering from evaporation, dissolution, photo-oxidation and biodegradation [38, 39].

**Fig 1:**
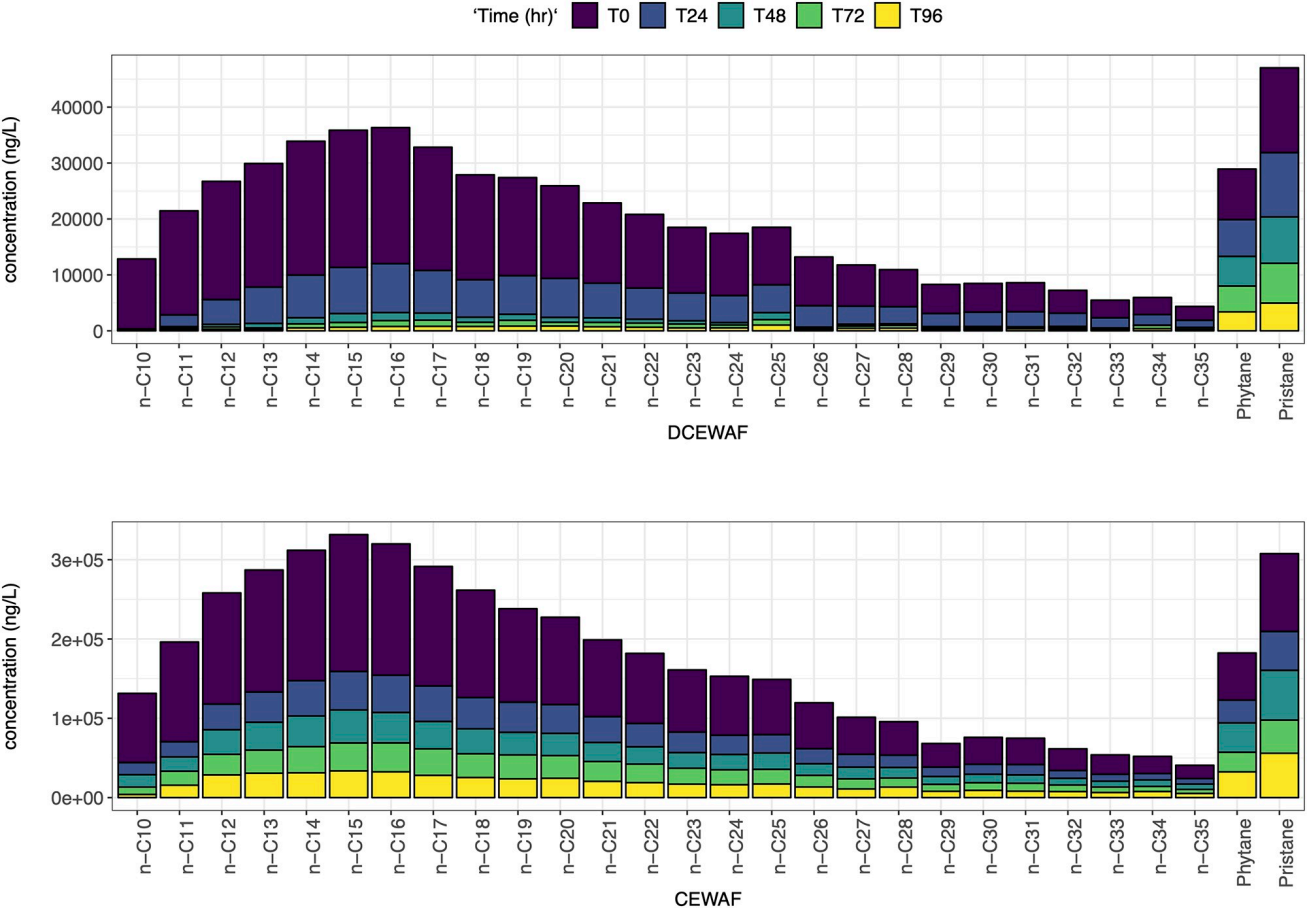
Concentration profiles of the surrogate oil: n-alkanes, pristane and phytane left at after 0, 1, 2, 3 and 4 days for Mesocosm 3.

**Fig 2:**
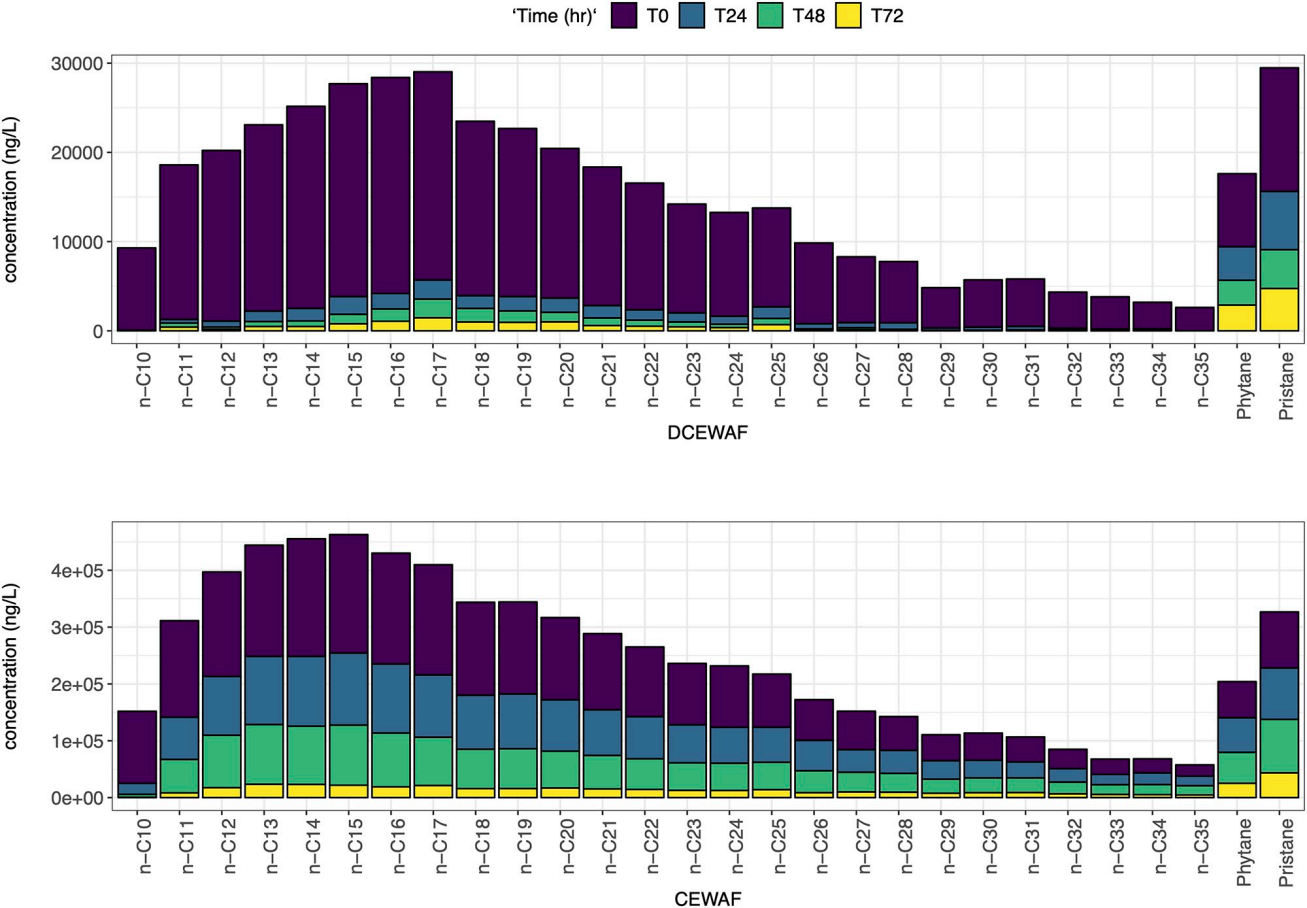
Concentration profiles of the surrogate oil: n-alkanes, pristane and phytane left at after 0, 1, 2, 3 and 4 days for Mesocosm 4.

The distribution of n-alkanes between n-C10 to n-C25 in M3 and M4 CEWAF and DCEWAF are shown in Figs 1 and 2. In both experiments n-alkane concentrations varied significantly within and between treatments. The n-alkane concentrations in the control treatments of M3 and M4 were close to or below the detection limits (<50 ng/L) for the duration of the experiments and so this data is not shown. The n-alkane distributions match the Macondo surrogate fingerprint from our analyses and reported analyses [18, 37] at time 0 days in both the DCEWAF and CEWAF (Figs 1 and 2) treatments. Low-molecular weight (LMW) n-alkanes (<C14) decreased rapidly in the DCEWAF and CEWAF treatments relative to the Macondo surrogate oil, indicating weathering processes such as evaporation or biodegradation. Microorganisms are able to degrade petroleum components in aerobic marine environments; preferentially degrading n-alkanes between n-C10 and n-C22 [18, 16]. This trend is apparent in the DCEWAF but less apparent in the CEWAF (Figs 1 and 2) treatment; most probably due to the higher concentrations of oil in the CEWAF and/or the presence of Corexit, requiring a longer time for the oil-degrading bacteria to alter the oil fingerprint [16]. In the DCEWAF treatment of M3 and M4, the bulk of the straight-chained n-alkanes decreased within the first 24 hr. In the case of the CEWAF treatments, the n-alkane concentrations remained high (Figs 1 and 2).

#### 3.3.1. Carbon preference index

To determine if the n-alkanes detected were biogenic (either algae or plant wax) or petrogenic, a carbon preference index (CPI) was calculated [40]. The relative abundance of odd versus even carbon-numbered n-alkanes is used to quantify plant wax contribution versus fossil fuel contamination [41, 42]. CPI values > 4 are related to biogenic n-alkanes, while CPI values ≤ 1 are evidence of fossil fuel contributions [43, 44]. Values that are <1 are considered to be of microbial origin [40]. The CPI equation from Romero et al. [45] follows:

CPI=Σ(oddCn)/Σ(evenCn)

The CPI calculated for the surrogate oil was 0.98 (Table 2). The CPI for WAF samples for M3 increased from 1.04 at the start of the experiment to 1.45 after 72 hours (Table 2) as a result of biodegradation of oil n-alkanes with a CPI of ~1 and the input of biogenic odd chained n-alkanes. In all other treatments for M3 and M4 the CPI was 1.0 with in the uncertainty of the measurements indicating that n-alkanes from the surrogate oil were the predominant source (Table 2). The CPI would change with time if n-alkanes were lost due to dissolution or evaporation as for example the lower the molecular weight the faster the removal. We do observe a faster decrease in n-C10 compared to the n-alkanes, but all alkanes concentrations are decreasing. Therefore, the disappearance of all n-alkanes is indicative of a predominant biodegradation over abiotic processes.

**Table 2:** Odd/even n-alkanes, nC_17_/pristane, nC_18_/phytane, and nC_17_/nC_18_ diagnostic ratios (standard deviation) for Macondo surrogate oil (Surr. Oil) and M3 and M4 with time in days.

		Days (d)	Odd/Even		nC_17_/Pr		nC_18_/Ph		nC_17_/nC_18_	
	Surr. Oil	NA	0.98	(±0.02)	1.92	(±0.02)	2.58	(±0.03)	1.21	(±0.01)
Mescosom 3	WAF	0	1.04	(±0.08)	1.01	(±0.25)	1.51	(±0.37)	1.59	(±0.31)
		1	1.30	(±0.18)	0.20	(±0.16)	0.20	(±0.05)	1.47	(±0.89)
		2	1.34	(±0.07)	0.30	(±0.10)	0.28	(±0.08)	1.35	(±0.15)
		3	1.45	(±0.25)	0.32	(±0.08)	0.62	(±0.35)	1.05	(±0.39)
		4	NA	NA	NA	NA	NA	NA	NA	NA
	DCEWAF	0	0.97	(±0.01)	1.45	(±0.04)	2.07	(±0.07)	1.18	(±0.01)
		1	1.00	(±0.04)	0.66	(±0.06)	1.02	(±0.10)	1.13	(±0.02)
		2	1.25	(±0.06)	0.15	(±0.00)	0.17	(±0.02)	1.43	(±0.21)
		3	1.11	(±0.14)	0.16	(±0.01)	0.16	(±0.03)	1.47	(±0.06)
		4	1.07	(±0.04)	0.16	(±0.01)	0.23	(±0.02)	1.05	(±0.11)
	CEWAF	0	0.97	(±0.02)	1.57	(±0.09)	2.30	(±0.08)	1.13	(±0.04)
		1	1.01	(±0.01)	0.93	(±0.08)	1.38	(±0.11)	1.20	(±0.06)
		2	1.00	(±0.03)	0.74	(±0.02)	1.15	(±0.10)	1.12	(±0.06)
		3	1.05	(±0.02)	0.85	(±0.06)	1.23	(±0.07)	1.16	(±0.06)
		4	0.97	(±0.04)	0.89	(±0.07)	1.34	(±0.12)	1.11	(±0.05)
Mesocosm 4	WAF	0	0.87	(±0.16)	0.6	(±0.12)	2.17	(±1.60)	1.53	(±2.08)
		1	1.00	(±0.01)	3.1	(±1.59)	2.54	(±0.89)	1.28	(±0.03)
		2	1.11	(±0.05)	8.3	(±4.50)	3.04	(±0.20)	1.32	(±0.06)
		3	1.06	(±0.03)	2.7	(±0.80)	1.57	(±0.11)	1.45	(±0.13)
	DCEWAF	0	1.00	(±0.05)	1.68	(±0.02)	2.39	(±0.01)	1.19	(±0.05)
		1	1.16	(±0.07)	0.28	(±0.06)	0.30	(±0.07)	1.56	(±0.08)
		2	1.25	(±0.14)	0.38	(±0.21)	0.45	(±0.25)	1.52	(±0.11)
		3	1.23	(±0.05)	0.69	(±0.15)	0.77	(±0.19)	1.37	(±0.02)
	CEWAF	0	0.99	(±0.00)	1.96	(±0.01)	2.57	(±0.05)	1.19	(±0.01)
		1	1.02	(±0.01)	1.10	(±0.29)	1.43	(±0.34)	1.19	(±0.09)
		2	1.03	(±0.01)	1.03	(±0.06)	1.42	(±0.07)	1.21	(±0.04)
		3	1.08	(±0.01)	0.49	(±0.08)	0.63	(±0.11)	1.37	(±0.03)

NA (not-available) data was a result of the expected low and variable concentrations in the WAF experiments of M3 at the end of this experiment.

#### 3.3.2. n-C17/Pr, n-C18/Phy and n-C17/n-C18 ratios

Biodegradation of oil is a complex and non-linear process involving simultaneous consumption of different oil classes at different rates [46], and by a mixture of multiple bacterial species [47, 48]. Alkane ratios to isoprenoids pristane and phytane were used to estimate biodegradation in the two experiments. The addition of chemical dispersants in seawater may complicate these processes as dispersants reduce the oil/water surface tension, resulting in smaller droplets that increase the area available for microbial colonization and biodegradation [49, 50, 51, 16].

The n-C17:Pr and n-C18:Phy ratios are well established indicators of oil biodegradation [8, 18, 52, 35] due to the branched isoprenoid hydrocarbons e.g. (Pr and Phy) being more resistant to biodegradation compared to the n-alkanes [8, 35]. The decreasing ratio of n-C17/Pr and n-C18/Phy in the WAF, DCEWAF and CEWAF of M3 and M4 is a reliable indication of biodegradation (Table 2). The ratio of n-C17/Pr in the DWH oil (MC252) ranged between 1.8–2.0 [18, 52, 29] and 2.2 for n-C18/Phy [53]. The ratios of n-C17/Pr, n-C18/Phy and Pr/Phy in the surrogate oil were 1.92, 2.59, and 1.62, respectively, similar to DWH oil. The n-C17/Pr and n-C18/Phy ratios observed in the WAF treatment were lower than Macondo surrogate oil suggesting partial microbial degradation of the oil during the ~24 hour preparation of the WAF. The ratio of n-C17/n-C18 in the Macondo surrogate oil (Table 2) of 1.21 is similar to the ratio of all treatments. Some treatments have ratios greater than 1.21 suggesting inputs of n-C17 from microbial biomass. These different rates may reflect differences in coastal (M4) versus open ocean (M3) microbial communities [16].

### 3.2 PAH concentrations

**Fig 3:**
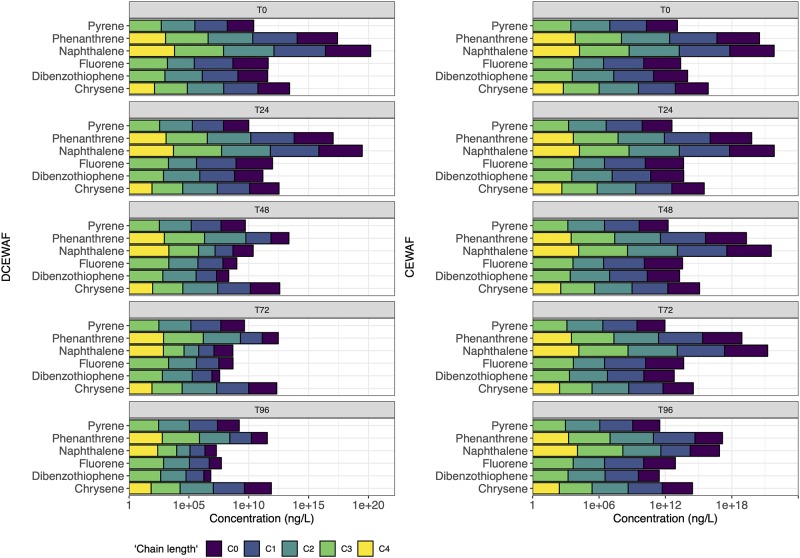
Concentration profiles of PAH left at after 0, 1, 2, 3 and 4 days for Mesocosm 3 (M3).

**Fig 4:**
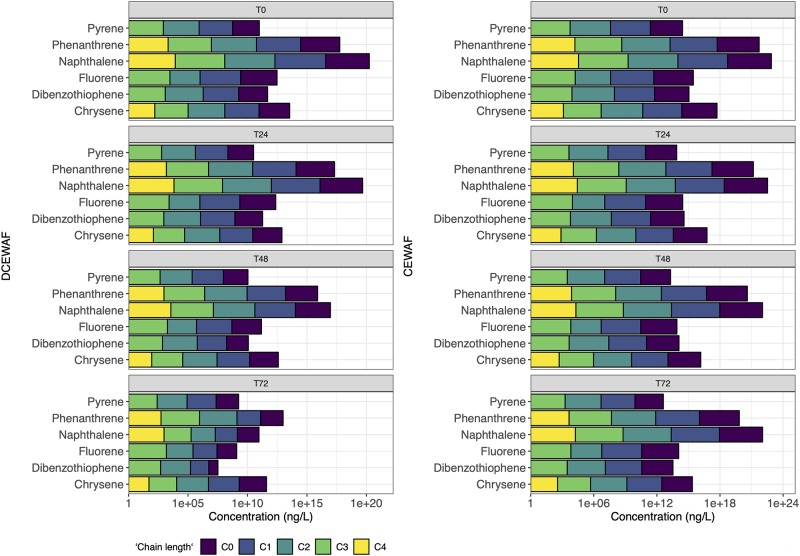
Concentration profiles of PAH left at after 0, 1, 2 and 3 days for Mesocosm 4 (M4).

PAH consisting of 41 individual PAH and their alkyl homologues were measured in the M3 and M4 experiments (Figs 3 and 4). Similar to the EOE values, PAH is lowest in WAF treatments and highest in CEWAF treatments (Table 2). While fluorescence spectrometry is a sensitive technique, GC/MS analysis can detect PAH when EOE is below the detection limit due to both a larger extraction volume for PAH (1–4 L vs 0.005–0.020L) and a final concentration step. This led to detection of PAH in the controls when no EOE was detected. The highest concentrations of PAH in the control was 1.4 ug/L, the initial conditions of Mesocosm 4, likely due to PAH present in the near-shore coastal water used in this mesocosm study.

The initial PAH concentrations (Table 1) were very similar in M3 and M4 experiments, except for CEWAF treatment of M4, where PAH concentration was almost twice as high as CEWAF treatment of M3 (Table 1). The change of individual PAHs concentrations is plotted in Fig 3 for M3 and Fig 4 for M4. In the natural environment, oil may be removed from the water column through various pathways: evaporation, photo-oxidation, sedimentation and biodegradation [54]. The absence of wind and low turbulence condition could lead to a much lower evaporation rate, even by orders-of-magnitude [55]. Photo-oxidation may also be occurring but was not specifically addressed and cannot be totally ruled out as a potential mechanism for PAH degradation in this study. Previous studies of biodegradation rates indicate large variation for different PAH’s. For example, it was found in another mesocosm study that the half-life of naphthalene was ~1 day while half-life of chrysene was determined to be 13 days [4]. The data from the mesocosm studies described here suggest a clear preference for the biodegradation of n-alkanes; however, the changes in PAH concentration may be the result of many other environmental parameters such as evaporation, sedimentation, photo-oxidation and/or biodegradation.

Quigg et al. [15] observed microbial exopolymers formation in all treatments within 24 hours, including the control. In this study it was suggested that the addition of oil and dispersant may further enhance bacterial growth and exopolymeric substance production, resulting in increased flocculation and formation of marine snow particulates [56, 57]. Oil, especially dispersed oil, readily undergoes adsorption to these particles [56] and can result in PAH removal through sedimentation. The biodegradation of the PAH likely continues during and after the sedimentation process as both highly weathered and unweathered oil was found in sediment samples [17].

Naphthalenes are the major PAH constituent at time 0 in WAF comprising 79%-83% of the total PAH, which is higher than the content in Macondo surrogate oil of 64%. Napthalenes in the DCEWAF ranged from 61% to 65% similar to the Macondo surrogate oil (Figs 3 and 4). Loss of naphthalenes from WAF and DCEWAF from both experiments within the first 48–72 hours is demonstrated (Figs 3 and 4) and is similar to other studies [2, 4], where LMW PAHs, especially naphthalenes, are more prone to rapid biodegradation [58, 59, 60]. However, in CEWAF treatments of both M3 and M4, the naphthalene percentage remains high (~45%-65%). In the first 72 hours of M3 and M4 CEWAF treatments (Figs 3 and 4), a slight increase of the percentage of naphthalene was observed perhaps due to heterogeneity or due to the loss of high molecular weight PAHs to the particulate phase [61]. At 72–96 hours in M3, the naphthalene percentage dropped, most probably due to biodegradation (Figs 3 and 4).

Alkylated PAH ratios of C2-dibenzothiophenes (DBT)/C2-phenanthrenes (D2/P2) and C3-DBTs/C3-phenanthrenes (D3/P3) have been used as indicators of biodegradation [62]. Each pair of alkylated DBT/phenanthrenes possess similar molecular weights, and DBT homologues are known to undergo certain biotransformation pathways [63]. A significant change in this ratio, whether positive or negative, indicates microbial degradation. For CEWAF treatments in M3 and M4 experiments, D2/P2 ratio stayed at an almost constant level of 0.25–0.29 and D3/P3 at 0.25–0.31. In WAF treatments, M3 showed a rapid increase beyond 48 hours, while M4 showed a relatively steady increase suggesting phenanthrenes are degraded faster than dibenzothiophenes [64]. The ratios in DCEWAF was similar to CEWAF–which stayed close to a constant–in the first 72 hours of M3 (0.21–0.28) and in M4 (0.22–0.28). However, at 96 hours of M3 there was a slight increase (0.39 for D2/P2 and 0.33 for D3/P3) similar to observations described by Olson et al. [62]. This indicates the biodegradation of petroleum aromatic hydrocarbons is occurring in the presence and absence of Corexit. It has been demonstrated that Corexit may alter the relative abundance of oil degrading microbes [16].

### 3.3 Half-lives

EOE, TPH, PAH, UCM, TR, and n-alkane concentrations decreased with time in both M3 and M4. The half-life of the average concentrations of these measurements are calculated assuming first order rate of decrease (Table 1). The half-lives of these measured oil components ranged from 0.5 to 8.1 days (Table 3). The half-lives for n-alkanes TR, UCM and TPH were not calculated for WAF, as the concentrations were at, or below the detection limit. The range of EOE WAF, DCEWAF and CEWAF half-lives for all treatments overlapped. This is due to the heterogeneity when oil and seawater are mixed and sampled when particles are present. However, the half-lives for PAH in M3 and M4 CEWAF are higher than other CEWAF treatments by a factor of 3 or more. In addition, the UCM and TPH half-lives for M3 UCM and TPH and M4 UCM and TPH are all above 2.8 days (Table 3). This is consistent with faster biodegradation of n-alkanes and lower molecular weight PAH than the rate for other petroleum components.

**Table 3:** Selected field and mesocosm estimated petroleum half-lives (days).

Field Studies	Half-Lives (days)	Reference
Range	0.4 to 25	Olson et al., 2017
**Mean**	**6.0**	Olson et al., 2017
**Median**	**3.0**	Olson et al., 2017
Gulf of Mexico	3.0	Hazen et al., 2010
Gulf of Mexico	2 to 16	Wang, et al., 2016
Gulf of Mexico	0.4 to 37	Thessen and North, 2017
**Mesocosm Studies**		
WAF	5.1	Gearing et al. 1979
WAF	1.3	Gearing and Gearing 1982
WAF	10.0	Gearing and Gearing 1982
WAF	2.5	Olson et al., 2017
WAF	13.8	Prince et al., 2013
WAF M2	1.4	Wade et al., 2017
WAF M3	1.2	this study
WAF M4	0.9	this study
**Mean**	**4.5**	
CEWAF	2.0	Olson et al., 2017
CEWAF	11.0	Prince et al., 2013
CEWAF M2	2.4	Wade et al., 2017
CEWAF M3	1.8	this study
CEWAF M4	1.5	this study
**Mean**	**3.7**	
DCEWAF M2	2.1	Wade et al., 2017
DCEWAF M3	3.2	this study
DCEWAF M4	1.4	this study
**Mean**	**2.2**	

The half-lives for the EOE reported here are compared to a summary of half-lives from other studies (Table 3). The half-lives for petroleum estimated from field studies ranged from 0.4 to 37 days (Table 3). During the DWH oils spill the half-life of the DWH oil was reported to be 3 days [49]. A biodegradation study using Macondo oil and indigenous Gulf of Mexico microbes at a temperature of <8 °C estimated half-lives of 0.6 to 9.5 days for n-C13 to n-C26 components of the oil while the more recalcitrant higher molecular weight PAH had half-lives of 60 days [51]. A summary of published rates of biodegradation of oils had half-lives that ranged from 0.5 to 260 days, with most in the range of a few days to a few weeks (see supplemental data [65]) It should be noted that the rapid loss of oil from suspended particles in the water column was reported to be slower after the particles reached the sea floor and particles with higher oil concentrations decayed even slower [66]. This suggest that once the particles reach the sediment biodegradation half-lives increase and agrees with the hydrocarbons’ fingerprints found as sediment oil residues [67]. Mesocosm WAF studies using a number 2 fuel oil [3, 68], a 42% by weight, evaporatively weathered DWH (MC252) oil [62], and Marlin platform oil (this study) half-lives ranged from 0.9 to 10 days. These half-lives are very similar in spite of the fact that these mesocosm water volumes were 0.15 L [62], 90L [7] and 1,300L [3]. The half-lives in the larger mesocosms ranged from 1.2 to 1.7 for July and September and was 10 days in March [68]. The difference was assumed to be due to evaporative losses as less hydrocarbons were associated with the sediments of the mesocosms [68]. In contrast field studies at a temperature of 5°C estimated half-lives of 3 days [48] which was higher than the 1.3 and 0.9 days respectively, for M3 and M4 WAF studies reported here which were at 20° C. The half-lives reported for WAF (13.8 days) and CEWAF (11 days) mesocosm studies with weather Alaskan North Slope crude oil at a concentration of 2.5 ppm (~ 2.3 ug/L) at 8°C in un-augmented New Jersey seawater [54] were in the higher end of the range (Table 3). The range of half-lives from field and mesocosm overlap and show the removal is on the order of days to a few weeks. The mesocosm half-life for CEWAF (Table 1) with the evaporatively weathered Deepwater Horizon oil and Corexit 9500A was 2.0 days [62]. The CEWAF half-lives for mesocosms prepared with the Marlin platform surrogate oil and the same dispersant ranged from 1.5 to 2.4 days with an average of 1.9 days. This indicates that regardless of scale (0.15 L verses 90L) and with similar oils the CEWAF half-lives are very similar to each other. The WAF and CEWAF half-lives are also in the range of the WAF half-lives with no indication that Corexit inhibits biodegradation at the concentrations of oil used [62].

### 3.4 Degradation sources

Abiotic degradation processes such as sedimentation dissolution, dispersion, or photooxidation [69] would be evident if the CPI changed considerably over time. However, there is clear trend of disappearance time as a function of chain length as shown with constant CPIs and the steep decrease of the n-C17/Pr and n-C18/Ph ratios over time (Table 2) that proves the dominance of biodegradation processes. In addition, abiotic processes would have affected the UCM half-life, which was not seen in our experiments (Table 1). The rapid disappearance of n-C10 and naphthalene (Figs 1, 2, 3 and 4) suggest that there could have been some evaporation [69] or photodegradation [70]; however, mesocosm tanks in all experiments were covered with glass lids and light conditions were of low intensity. In addition, microbial preference for short-chain hydrocarbons was documented using the nC17/nC18 ratio (Table 2).

The oil and oil plus dispersant mixtures were prepared in the dark and hence, photooxidation during this process can be discarded. Parallel studies performed by other members of the ADDOMEx consortia during our mesocosm experiments found that the low light conditions and the rapid proliferation of hydrocarbon-degraders suggest biological activity as the primary cause of oil degradation [71, 16]. Additionally, previous studies reported that alkanes from oil spill in surface water did not suffer photodegradation after being exposed to natural light for a 12 hour period [69]

MOS was deposited to the bottom of the mesocosm tanks as a flocculants layer confirming that biotic enhanced sedimentation from EPS production was occurring [15]. The flocculation of MOS can be considered a sedimentation process as well as biological, where entrapped oil droplets are both physically removed from the water column and biodegraded by a plethora of microorganisms [17].

## Conclusions

Our controlled, replicate ~100 L mesocosm studies expand our knowledge for surface waters of the rates of these processes under varying environmental conditions with and without dispersant. Our conclusion is that for surface waters dispersant do not significantly affect the half-lives of the oil components. Thus, if the response goal is to keep oil from coastal areas then the use of dispersant is warranted.

Our study proves that baffled recirculation tanks are capable of generating replicable concentrations of large volumes of oil and oil plus dispersant mixtures. The latter provides an alternative to the CROSERF method, which can only produce small volumes of the oil mixtures. Therefore, the baffled recirculation system opens the possibility to replicable large-scale studies at a lower cost; saving time and production effort.

The variability of oil concentrations in the WAF, DCEWAF and CEWAF mesocosms was due to the high hydrophobicity of oil and provides a challenge in producing and sampling a stable oil/water mixture. A variety of factors affect the homogeneity of the mixtures. Firstly, oil droplets associated with the marine snow when MOS was present contributes to heterogeneity. Secondly, the addition of Corexit increased the number of small oil droplets thereby increasing the oil concentration and influencing the variability in the measurements. In addition, weathering processes such as evaporation, sedimentation, photo-oxidation and biodegradation may occur at different rates within the triplicate treatments.

The half-lives of most petroleum components in mesocosms studies and in the marine environment are a few days. In this study, the preferential degradation of n-C17 compared to pristane and n-C18 compared to phytane confirms biodegradation is occurring in the WAF, DCEWAF and CEWAF treatments. Components of the oil that are more resistant to biodegradation, such as UCM, have longer half-lives in the WAF, DECWAF and CEWAF. The addition of nutrients ensured that microbial growth was not nutrient limited. There is no clear-cut evidence that the nutrients which were added, accelerated the degradation of oil as half-lives were in the range of other mesocosm and field studies. Both mesocosm experiments, open-ocean water experiments (M3) and coastal water (M4) had comparable hydrocarbon half-lives. In addition, the half-lives for DCEWAF and CEWAF treatments are in the same range as WAF with just oil added and no dispersant indicating that the addition of dispersant had no measurable effect on the half-lives of the oil. These mesocosm experiment indicate biodegradation of oil is occurring which is confirmed by microbial results from these same mesocosm studies [16].

The linear disappearance of n-alkanes and preference of n-C17 and n-C18 over Pr and Phy, plus the constant CPIs and half-lives of the oil compounds, strongly suggest that microbial biodegradation was the most prominent source of oil degradation. Low light conditions and proliferation of hydrocarbon-degraders measured by other members of our team are in agreement with our findings [69, 16].
